# Molecular phylogenetics and comparative modeling of HEN1, a methyltransferase involved in plant microRNA biogenesis

**DOI:** 10.1186/1471-2148-6-6

**Published:** 2006-01-24

**Authors:** Karolina L Tkaczuk, Agnieszka Obarska, Janusz M Bujnicki

**Affiliations:** 1 Laboratory of Bioinformatics and Protein Engineering, International Institute of Molecular and Cell Biology, Trojdena 4, 02-109 Warsaw, Poland; 2 Institute of Technical Biochemistry, Technical University of Lodz, Stefanowskiego 4/10, 90-924 Lodz, Poland; 3 Institute of Molecular Biology and Biotechnology, Adam Mickiewicz University, Umultowska 89, 61-614 Poznan, Poland

## Abstract

**Background:**

Recently, HEN1 protein from *Arabidopsis thaliana *was discovered as an essential enzyme in plant microRNA (miRNA) biogenesis. HEN1 transfers a methyl group from S-adenosylmethionine to the 2'-OH or 3'-OH group of the last nucleotide of miRNA/miRNA* duplexes produced by the nuclease Dicer. Previously it was found that HEN1 possesses a Rossmann-fold methyltransferase (RFM) domain and a long N-terminal extension including a putative double-stranded RNA-binding motif (DSRM). However, little is known about the details of the structure and the mechanism of action of this enzyme, and about its phylogenetic origin.

**Results:**

Extensive database searches were carried out to identify orthologs and close paralogs of HEN1. Based on the multiple sequence alignment a phylogenetic tree of the HEN1 family was constructed. The fold-recognition approach was used to identify related methyltransferases with experimentally solved structures and to guide the homology modeling of the HEN1 catalytic domain. Additionally, we identified a La-like predicted RNA binding domain located C-terminally to the DSRM domain and a domain with a peptide prolyl cis/trans isomerase (PPIase) fold, but without the conserved PPIase active site, located N-terminally to the catalytic domain.

**Conclusion:**

The bioinformatics analysis revealed that the catalytic domain of HEN1 is not closely related to any known RNA:2'-OH methyltransferases (e.g. to the RrmJ/fibrillarin superfamily), but rather to small-molecule methyltransferases. The structural model was used as a platform to identify the putative active site and substrate-binding residues of HEN and to propose its mechanism of action.

## Background

MicroRNAs (miRNAs) are small (~22 nt), single-stranded, noncoding RNAs that have recently emerged as important regulatory factors during growth and development in Eukaryota. To date, miRNAs were described in animals, plants, and viruses (reviews: [1-3]). miRNAs are processed from longer precursor RNAs transcribed by RNA polymerase II that form stem-loop structures, in which the mature miRNAs reside in the stems. In animals, long primary transcripts (pri-miRNAs) are first cropped in the nucleus by an RNase-III homolog Drosha to release the hairpin intermediates (pre-miRNAs) in the nucleus. Following their export to the cytoplasm, pre-miRNAs are subjected to the second processing step, which is carried out by another RNase III homolog Dicer. In plants that lack Drosha, it has been suggested that miRNA processing is executed by Dicer-like protein 1 (DCL1, also called CARPEL FACTORY or CAF) (reviews: [4,5]). miRNAs down-regulate gene expression by binding to complementary mRNAs and either triggering mRNA elimination or arresting mRNA translation into protein. Thus far, miRNAs have been implicated in the control of several pathways, including developmental timing, haematopoiesis, organogenesis, apoptosis, cell proliferation and possibly even tumorigenesis (reviews: [6-8]). However, the mechanisms of miRNA generation and function are still poorly understood and the molecular details are only beginning to be revealed.

*HEN1 *was identified as a gene that plays a role in the specification of stamen and carpel identities during the flower development in *Arabidopsis thaliana *[9]. Mutations in *HEN1 *resulted in similar defects to those observed for mutations in *CAF*, suggesting that they are both involved in miRNA metabolism [10]. Recently, it was found that the product of *HEN1 *is a methyltransferase (MTase) that acts on miRNA duplexes *in vitro *and methylates the last nucleotide of both strands in the substrate [11]. It was found that the methylation by HEN1 protects plant miRNAs against the 3'-end uridylation and the subsequent degradation [12]. Both the 2'-OH and 3'-OH groups of ribose on the last nucleoside were found to be essential for methylation by the HEN1 protein, hence they are both considered as the possible methylation sites, they may also play a crucial role in the process of substrate recognition [11]. The 2'-OH group is the most commonly methylated target in RNA, while 3'-methylated ribonucleosides have not been identified [13]. However, it remains to be determined which of the OH groups of the last nucleoside of the miRNA/miRNA* duplex is the target of methylation by HEN1. Of note, HEN1 and its homologs analyzed in this article are completely unrelated to a human gene HEN1 that encodes a 20-kDa neuron-specific DNA-binding polypeptide (pp20HEN1) with the basic helix-loop-helix (bHLH) motif.

HEN1 is a long protein (942 aa), which was found to comprise a putative double-stranded RNA-binding motif (DSRM) in the very N-terminus and a C-terminal domain (CTD, aa ~694–911), which exhibits significant similarity to a group of uncharacterized protein from bacteria, fungi, and metazoa [10]. These proteins are however much shorter – they lack the DSRM and the long central region of HEN1. HEN1-CTD was found to be related to the Rossmann-fold MTase (RFM) superfamily, suggesting that it is responsible for the RNA MTase activity of this protein [14]. It is noteworthy that sequences of HEN1 and its homologs are so strongly diverged from other proteins that initially HEN1 was not recognized as a MTase homolog when it was discovered [10]. Thus, apart from generic features common to all MTases, the molecular mechanism of specific interactions of HEN1 with its substrate RNA remains unknown. In particular, the three-dimensional structure, the identity of potential catalytic and substrate-binding residues, and the phylogenetic origin of HEN1-CTD have not yet been inferred. We have therefore carried out bioinformatics analyses to collect the possibly most complete set of HEN1 orthologs in current sequence databases as well as to identify closest paralogs amongst MTases with known structure and mechanism of action. The results were used to construct a tertiary model of the catalytic domain of HEN1 and to predict the architecture of the substrate-binding region and the active site.

## Results and discussion

### Sequence analyses of HEN1

In order to identify orthologs of *A. thaliana *HEN1, we used its full-length sequence as a query to search the non-redundant (nr) protein database using PSI-BLAST [15] as well as genomic databases using tBLASTn [16]. A complete homologous sequences with significant similarity to the entire query was found only in *Oryza sativa *(gi: 50510095). We also searched the EST and genomic databases using tBLASTn [16] and found sequences from several different plant species that covered various segments of the query, but from which we could not assemble any contiguous fragment that would cover the full-length protein. Only the HTG sequence from *Lotus corniculatus *var. japonicus (gi: 17736840) displayed similarity to the entire query sequence, but we decided to omit it from further analyses due to uncertainties in positions of intron-exon boundaries (data not shown).

To identify domains in the primary structure of HEN1, we carried out an RPS-BLAST search of the CDD database of conserved domain alignments [17], which confirmed the presence of the N-terminal DSRM, albeit with low score (e-value 0.73, only 67.6% aligned) and the C-terminal RFM domain (aa 690–940; best match to the UbiG MTase family, e-value 6*10^-05^), but did not reveal any new domains in the large central region. Therefore, we divided the HEN1 sequence into a set of overlapping sequence fragments < 500 aa and submitted it to the GeneSilico protein structure prediction MetaServer [18] to carry out predictions of secondary structure, protein order/disorder and possible three-dimensional folds (see Methods for details). Fragments of 100–200 aa with apparent similarity to conserved domains were resubmitted as individual jobs.

**Figure 1:**
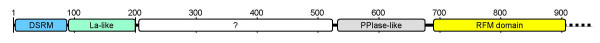
**Primary structure of *A. thaliana *HEN1**. Domains homologous to known protein families are indicated by color boxes. The unassigned region (with no detectable homology to other families or structures) is indicated by a question mark. Dashed lines indicate the region of predicted disorder.

Figure 1 summarizes the results of the primary structure analysis of HEN1. The fold-recognition (FR) analysis supported the presence of the DSRM in the N-terminus (aa 1–90) with significant scores (e.g. INBGU score 128.54, PCONS2 score 1.974). Interestingly, immediately next to the C-terminus of the DSRM (aa 91–200) we detected another putative RNA-binding domain, namely the La domain (a member of the wHTH fold) (PCONS2 score: 1.669). It is noteworthy that the founding member of the La family specifically recognizes the 3'-OH group of a poly-U end of RNA polymerase III transcripts [19], suggesting that the La domain in HEN1 could be involved in the recognition of the 3'-OH group in the miRNA/miRNA* duplex. In the region preceding the catalytic domain (aa ~530–680), we identified a domain homologous to the peptidyl prolyl *cis-trans *isomerase (PPIase) family, confidently aligned by all FR servers to the FK506-binding protein (FKBP) structure (e.g. PCONS2 score: 1.629). PPIases alter the orientation of peptide chains at proline residues, thereby aiding proper protein folding [20]. FKBP also participates in silencing and exhibits histone chaperone activity, but its PPIase domain was found not to be essential for this function [21]. The FKBP-like superfamily includes also the C-terminal domain of the GreA transcript cleavage factor, a protein that acts by inducing hydrolytic cleavage of the transcript within the RNA polymerase, followed by release of the 3'-terminal fragment [22]. However, it has been inferred that GreA binds RNA using its N-terminal domain, while the C-terminal domain participates in protein-protein interactions with the polymerase [23]. The predicted PPI-like domain in HEN1 lacks the typical PPIase active site, in particular the aromatic residue that provides a platform for binding of the proline and Tyr residue that coordinates the substrate (e.g. W59 and Y82 in FKBP12). Thus, the function of the PPI-like domain in HEN1 remains to be determined experimentally. Finally, we predict that the central region of HEN1 (aa 201–529) exhibits a pattern of helices and strands typical for well-folded globular domains; however, we were unable to identify its relationship to any known structures or conserved protein families.

To study the origin of the HEN1 enzyme, we carried out additional searches of the non-redundant sequence database using only HEN1-CTD (aa 694–911), with a stringent e (expectation) value threshold of 10^-20^. The search converged in the 4^th ^iteration, revealing a family of sequences with well-conserved regions along the entire sequence. All sequences with scores below that threshold were reported with significantly shorter alignments and a preliminary visual analysis suggested that they lacked many of the residues apparently conserved among the close homologs of HEN1, they were also annotated as involved in distinct processes (typically – methylation of quinones), hence they were regarded as potential paralogs.

**Figure 2:**
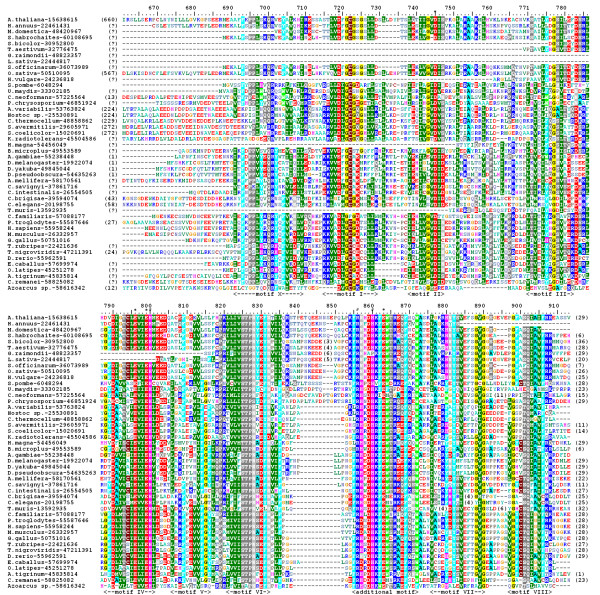
**Sequence alignment of the HEN1 family**. Amino acids are colored according to the physico-chemical properties of their side-chains (negatively charged: red, positively charged: blue, polar: magenta, hydrophobic: green. Residues conserved in > 50% sequences are highlighted. Putative catalytic residues are indicated by "*", putative RNA-binding residues are indicated by "#". Predicted secondary structure elements are shown below the alignment. Terminal extensions and non-conserved insertions have been removed for clarity (the number of omitted amino acids is indicated). Conserved motifs common to most of RFM enzymes are indicated by Roman numerals, the motif characteristic for the HEN1 family is also indicated.

For the purpose of analyzing the orthologs of HEN1-CTD, all sequences reported with the e-values better than the threshold of 10^-20 ^were retrieved and automatically aligned using MUSCLE [24]. This initial alignment was refined manually (as described in Methods) to remove redundant sequences. Members of sequences from different phylogenetic lineages (see below) were used as queries in additional tBLASTn searches of the dbEST database and of finished and unfinished genomes, to identify additional members of the HEN1 family and also to refine some of the sequences from the initial alignment, which seemed to exhibit deletions due to overlooked exons etc. The final set comprised 46 sequences, including 25 members from Metazoa, 4 from Fungi, 11 from Viridiplantae, and 6 from Bacteria. Figure 2 shows the final multiple sequence alignment (MSA) of the HEN1 family, which was refined based on the results of structure prediction and evaluation of the sequence-structure fit on the three-dimensional level (see below).

To identify closest paralogs (and potential ancestors) of HEN1, we converted the multiple sequence alignment of the HEN1 family into a profile-Hidden Markov Model (HMM) using HHpred [25] and we compared it with similar profile-HMMs pre-calculated for protein families collected in the Clusters of Orthologous Groups (COG) database [26]. Interestingly, HHpred analysis suggested that the closest relatives of HEN1 are not MTases acting on nucleic acids, but enzymes acting on small molecules. The top three matches that obtained significantly higher similarity scores than other families, were: COG2227 (UbiG, "2-polyprenyl-3-methyl-5-hydroxy-6-methoxy-1,4-benzoquinol methylase"; reported with e-value: 3.1*10^-24^), COG2230 ("cyclopropane fatty acid synthase and related methyltransferases", reported with probability e-value:1.8*10^-21^), and COG2226 (UbiE, "methylase involved in ubiquinone/menaquinone biosynthesis", reported with e-value: 6.9*10^-20^). The fourth match, with already significantly lower score was COG4106 ('trans-aconitate MTase', e-value: 8.3*10^-16^). The best-scoring nucleic acid MTase family on the list of HEN1 homologs was found only on the fifth position (COG2519, "GCD14 tRNA (1-methyladenosine) methyltransferase and related methyltransferases"), with e-value 1.2*10^-12^. It is remarkable that no known ribose MTase families were reported at the top positions of the ranking.

**Figure 3:**
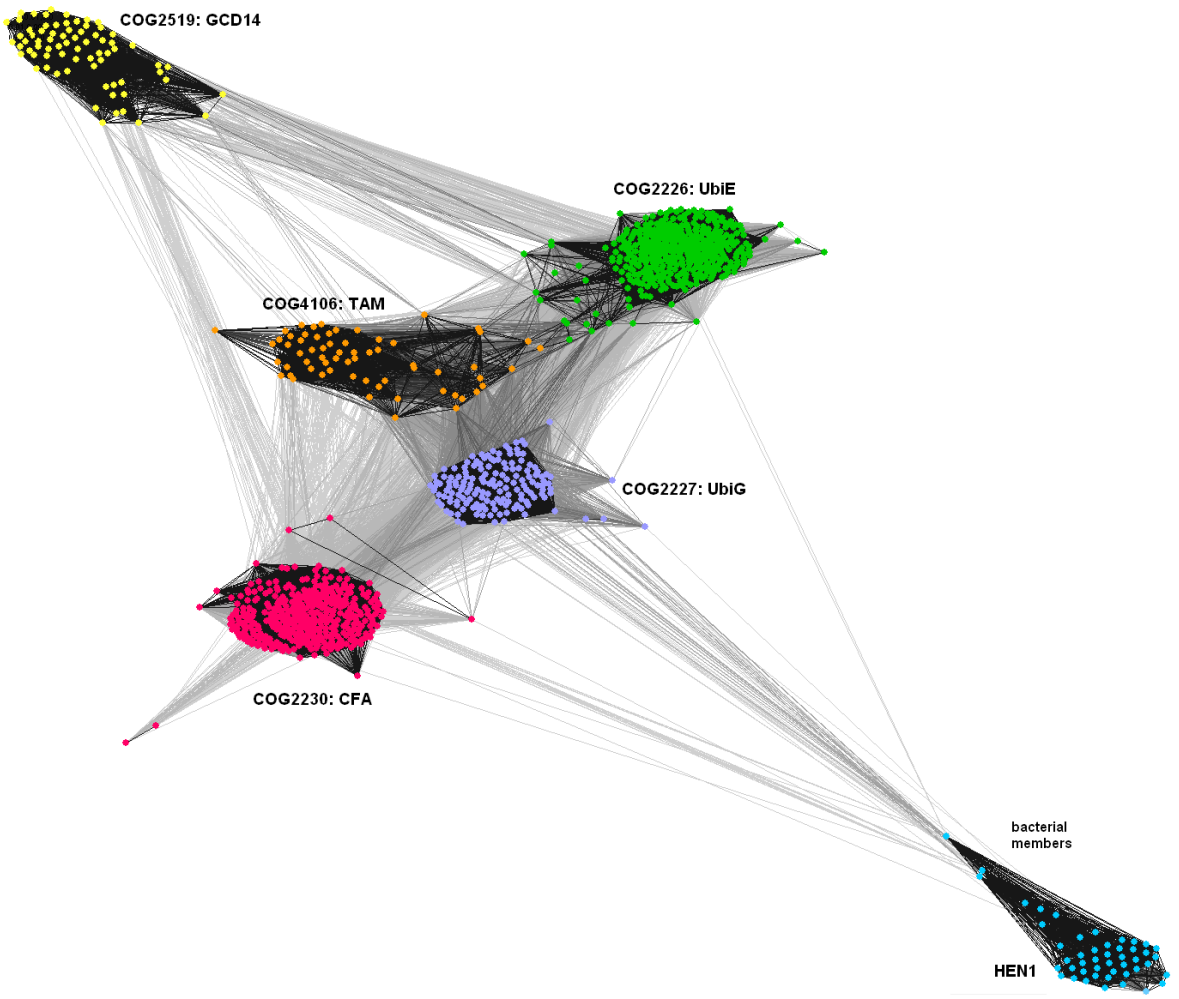
**2D cluster analysis of full-length sequences of the HEN1 family and most similar COGs**. Line coloring reflects BLAST P-values; dark lines represent pairwise connections with very low P-values (high similarities), lighter lines those with P-values closer to the cutoff (10^-5^). Members of each COG are indicated with a different color.

From the results of the aforementioned PSI-BLAST search queried with the HEN1-CTD we retrieved additional sequences reported with e-values 10^-20^-10^-30^. We also carried out PSI-BLAST searches for all five aforementioned closest paralogs of HEN1 to retrieve all or at least a substantial fraction of members of these lineages. We combined all these sequences with HEN1 orthologs and after removing duplicates found by more than one search, we clustered them based on the pair-wise BLAST similarity scores using CLANS [27]. We tried different P-value thresholds and found that the value of 10^-5 ^produced best-resolved sequence "clans" corresponding to different COGs (with very strong connections within each clan and preferred connections between a few, but not all clans). Figure 3 shows that the HEN1 clan is connected most strongly to the UbiG clan (COG2227), mostly via the bacterial members of the HEN1 family. In agreement with the results, the two next neighbors, closely related to UbiG, are UbiE (COG2226) and CFA (COG2230), while TAM (COG4106) and tRNA:m^1^A MTases (COG2519) are evidently more distantly related. Thus, the catalytic domain of HEN1 appears to be a close relative of quinone MTases and related small-molecule modifying enzymes (with UbiG being the closest detectable paralogous family) and only a remote homolog of other nucleic acid MTases.

### Phylogenetic analysis of HEN1-CTD

**Figure 4:**
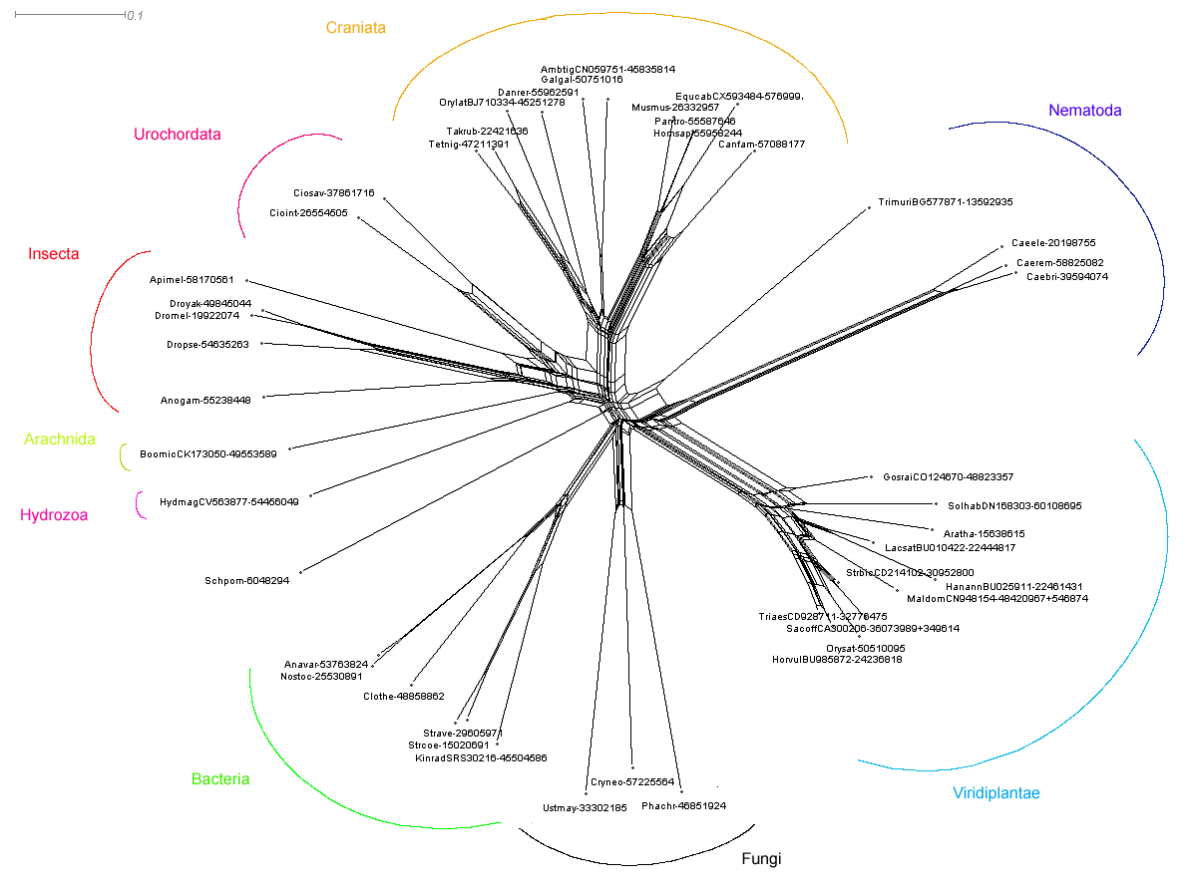
**Results of split-decomposition analysis of the HEN1 family**. 1000 bootstrap replicates were generated; the average reliability of edges in the split graph is 88%. According to SPLITSTREE, the fit index of the graph is 96.89%. Names at the brackets of the split graph represent names of taxons corresponding to monophyletic lineages. Edges are drawn to scale, with the bar indicating 0.1 aa replacements per site (estimated using the JTT model).

The distribution of HEN1 family members among different phyla is quite unusual, suggesting that interesting insights into its origin may be obtained from the inference of its evolutionary history. Thus, based on the alignment we calculated phylogenetic trees of the HEN1 family using several different methods. Unfortunately, all traditional approaches, including the neighbor-joining, maximum parsimony, and maximum likelihood methods failed to produce a tree with well-resolved branches and without erratic grouping of sequences from distantly related species, possibly due to the long branch attraction (data not shown). Therefore, we decided to study relationships within the HEN1 family using methods that infer a "fuzzy" picture of possible evolutionary connections. Figure 4 shows the phylogenetic tree of the HEN1 family generated with SPLITSTREE, using the split decomposition method [28]. The largest branch comprises most of the Metazoan members of the HEN1 family (including Craniata, Urochordata, Insecta, Arachnida, and Hydrozoa) with the exception of Nematoda. The branch comprising members of three *Caenorhabditis *species (*C. elegans*, *C. remanei*, and *C. briggsae*) groups together with Viridiplantae, but seems to be connected to the main Metazoan cluster by the intermediate location of *Trichuris muris*. Other main branches are formed by Viridiplantae, Bacteria, and Fungi, with the exception of *Schizosaccharomyzes pombe*, whose position is unresolved.

**Figure 5:**
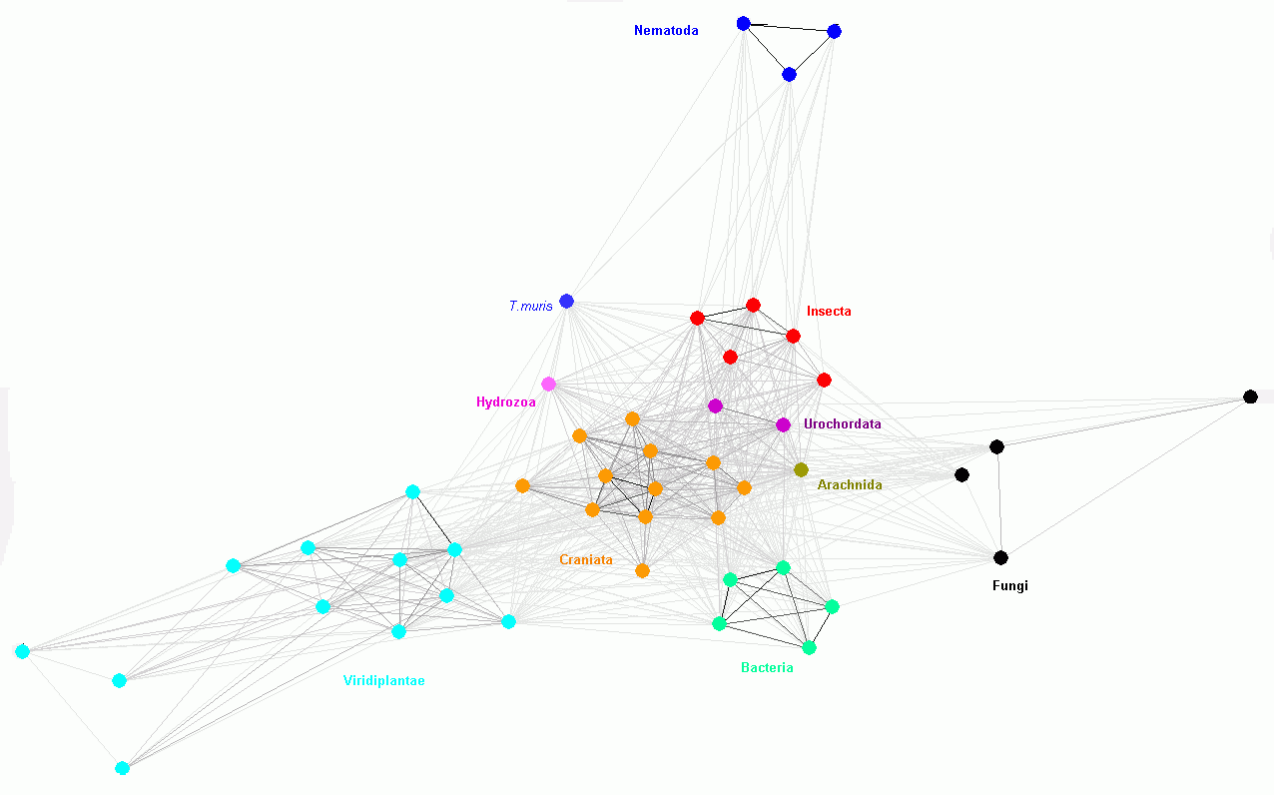
**2D cluster analysis of catalytic domains of the HEN1 family**. Members of each taxon are indicated with different colors.

We have also analyzed similarities within the HEN1 family by clustering them with CLANS [27]. We have experimentally found that the P-value threshold of 10^-22 ^produced qualitatively best results. More stringent values caused disconnection of the most diverged sequences, while more permissive values, such as 10^-5 ^used earlier for the analysis of relation of HEN1 to other COGs, caused over-compaction of the whole dataset into a single cluster with only a few outliers. Figure 5 shows a representative 2D projection of "sequence clans" obtained after several independent minimizations, starting with random distribution of sequences. This analysis reproduced all the groupings corresponding to taxons outlined by the split decomposition method, but also revealed additional meaningful associations. Craniata, Urochordata, Insecta, Hydrozoa, and Arachnida form a single central cluster, surrounded by clusters of Viridiplantae, Fungi, Nematoda, and Bacteria. Interestingly, this analysis revealed association of *S. pombe *SPBC336.05c with other fungal members of the HEN1 family (*C. neoformans*, *U. maydis *and *P. chrysosporium*). The nematode *T. muris *appears much closer to the central cluster than sequences from *Caenorhabditis*. This suggests that the *Caenorhabditis *branch underwent accelerated evolution and explains that its peculiar grouping with Viridiplantae in the SplitsTree reconstruction (Figure 3) may be due to the "long branch attraction" artifact. Bacteria form a well-resolved, dense cluster, located relatively close to the central metazoan cluster, but with connections also to fungal and plant clusters. Finally, the plant cluster comprises the main part (including *A. thaliana *HEN1) and three outliers, *Lactuca sativa *(GI:22444817), *Gossypium raimondii *(GI: 48823357), and *Triticum turgidum *(GI: 39729852).

The relationships between different eukaryotic lineages of HEN1 are in general agreement with the topology of the "Tree of Life". In Fungi they are present both in Basidiomycota (e.g. *U. maydis*) and Ascomycota (e.g. *S. pombe*), but they appear to have been lost from many lineages, e.g. Saccharomycotina. It is noteworthy that HEN1 orthologs could not be detected in Archaea or in primitive Eukaryota with fully sequenced genomes, such as Alveolata (e.g. *Plasmodium*) or Euglenozoa (e.g. *Trypanosoma*). On the other hand, the distribution of HEN1 homologs in Bacteria is very limited and quite erratic (only in Firmicutes – *Clostridium *and *Streptococcus*, Cyanobacteria – *Nostoc *and *Anabaena *and Actinobacteria – *Kineococcus radiotolerans*). This distribution would suggest that HEN1 originated in the common ancestor of Eukaryota, before the divergence of the Viridiplantae and Metazoa/Fungi branches, and has been transferred horizontally to Bacteria. However, the sequences of bacterial members of the HEN1 family appear to be more similar to the closest paralogous family UbiG than the eukaryotic members (Figure 3). This suggests that HEN1-CTD could have evolved in Bacteria by duplication of the UbiG-encoding gene and neofunctionalization of the second copy and only then was horizontally transferred to the ancestor of contemporary Eukaryota. In order to fully understand the origin of HEN1, it will be useful to characterize the molecular function of the short forms (lacking the N-terminal extensions of *A. thaliana *HEN1) from Bacteria as well as from other eukaryotic species (in particular animals and fungi).

### Structure prediction of the HEN1-CTD

In the absence of an experimentally determined protein structure, comparative modeling may provide a structural platform for the investigation of sequence-structure-function relationships. This technique requires a homologous template structure to be identified and the sequence of the modeled protein (a target) to be correctly aligned to the template. The C-terminal catalytic domain of HEN1 showed distant similarity to many different structures of class-I MTases in standard database searches. It is known, however, that despite the common fold and conserved cofactor-binding site, different subfamilies of MTases exhibit significant differences in the architecture of their substrate-binding pocket and the active site (e.g. ref. [29,30]). Thus, modeling of HEN1 based on a randomly selected MTase structure could introduce large errors in the functionally most important parts of the protein and mislead the structure-based functional predictions.

In order to identify the optimal set of template structures for modeling of HEN1, we used the fold-recognition (FR) approach, which allows to assess the compatibility of the target sequence with the available protein structures based not only on the sequence similarity, but also on the structural considerations (match of secondary structure elements, compatibility of residue-residue contacts, etc.). As mentioned earlier, the sequence of HEN1 CTD was therefore submitted to the GeneSilico protein fold-recognition metaserver [18]. As expected, all FR methods reported RFM structures as the potentially best templates. Interestingly, none of them reported any of the known RNA:2'-OH MTase structures from the RrmJ/fibrillarin superfamily [31] or actually, any known RNA or DNA MTases, on top positions of the ranking. Instead, all FR algorithms suggested that the potentially best templates for modeling of HEN1 (i.e. its closest homologs among proteins of known structure) are either known small-molecule MTases or uncharacterized proteins from the structural genomics projects, which show strongest similarity to small-molecule MTases. In particular, PDB-BLAST reported 1 kpg (a mycolic acid cyclopropane synthase Cmaa1 from *Mycobacterium*) with the score of 2*10^-42^, FFAS [32] reported 1xxl (an uncharacterized protein YcgI from *Bacillus subtilis*) with the score of: -44.1, mGENTHREADER [33] reported 1xxl with the score of 0.949, SPARKS [34] reported 1vl5 (an uncharacterized protein Bh2331 from *Bacillus halodurans*) and 1y8c (an uncharacterized, predicted MTase from *Clostridium acetobutylicum*) with the score of -4.42 (these scores are not normalized as each server uses a different evaluation system; see the individual references for details). Ultimately, the consensus server Pcons2 [35] assigned highest scores (2.673-2.42) to the small-molecule MTase structures 1xxl, 1vl5, and 1 kpg, as potentially best templates for modeling of HEN1. This result is in very good agreement from the profile-HMM analysis, which suggested that HEN1 is most closely related to small-molecule MTase families, including those with unknown structures such as UbiE and UbiG (which are thus unavailable for detection by the structure-based FR). Thus, bioinformatics analyses strongly suggests that HEN1 CTD exhibits sequence and structural features characteristic for the "small molecule" branch of the MTase superfamily.

### Comparative modeling of the HEN1-CTD

A comparative model of HEN1 was constructed based on the alignments reported by fold-recognition methods, using the "FRankenstein's Monster" approach [36](see Methods). The C-terminal residues 912–942 were predicted to be disordered by DISOPRED [37] and PONDR [38] (data not shown), and therefore they were omitted from the analysis. The final model comprising residues 694–911 was constructed by iterating the homology modeling procedure (initially based on the raw FR alignments to the top-scoring templates 1 kpg, 1vl5, 1y8c, and 1xxl), evaluation of the sequence-structure fit by VERIFY3D, merging of fragments with best scores, and local realignment in poorly scored regions. Local realignments were constrained to maintain the overlap between the secondary structure elements found in the MTase structures used as modeling templates, and predicted for HEN1. This procedure was stopped when all regions in the protein core obtained acceptable VERIFY3D score (>0.3) or their score could not be improved by any manipulations, while the average VERIFY3D score for the whole model could not be improved.

**Figure 6:**
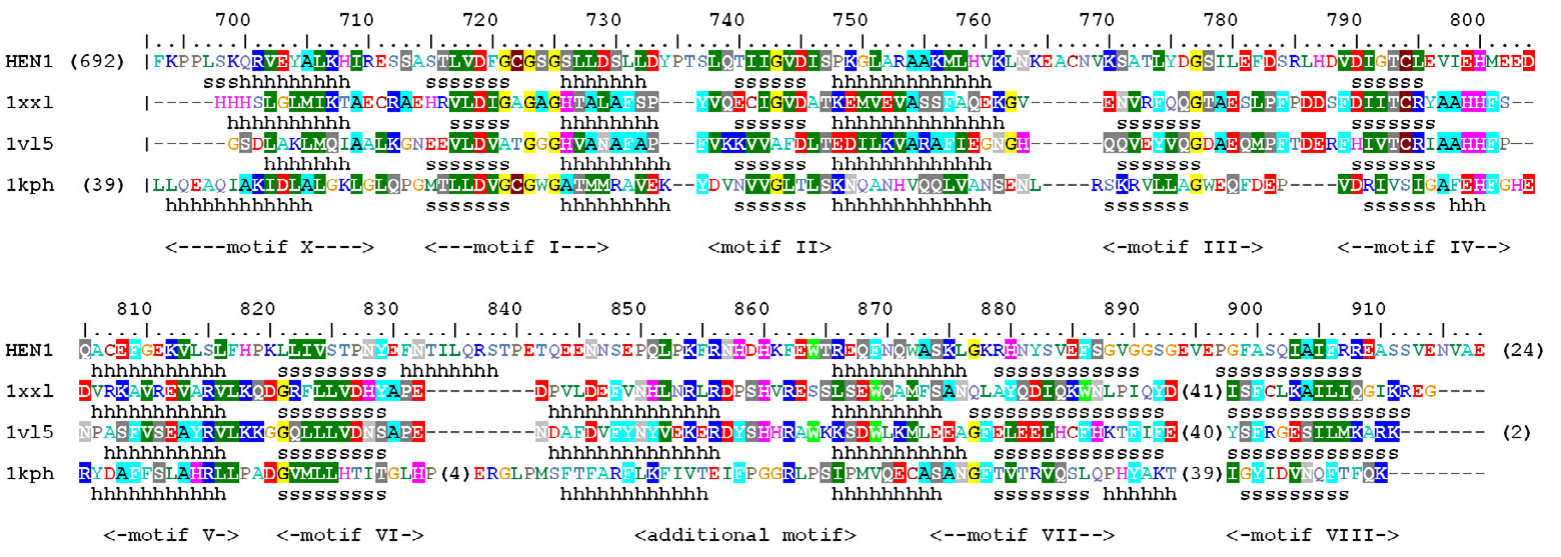
**Fold-recognition alignment between sequences of HEN1 and its most preferred templates**. Amino acids are colored according to the physico-chemical properties of their side-chains (negatively charged: red, positively charged: blue, polar: magenta, hydrophobic: green. Pairs of residues conserved between HEN1 and the templates are highlighted. The pattern of secondary structures experimentally determined for the templates and predicted for HEN1 is shown below each sequence.

**Figure 7:**
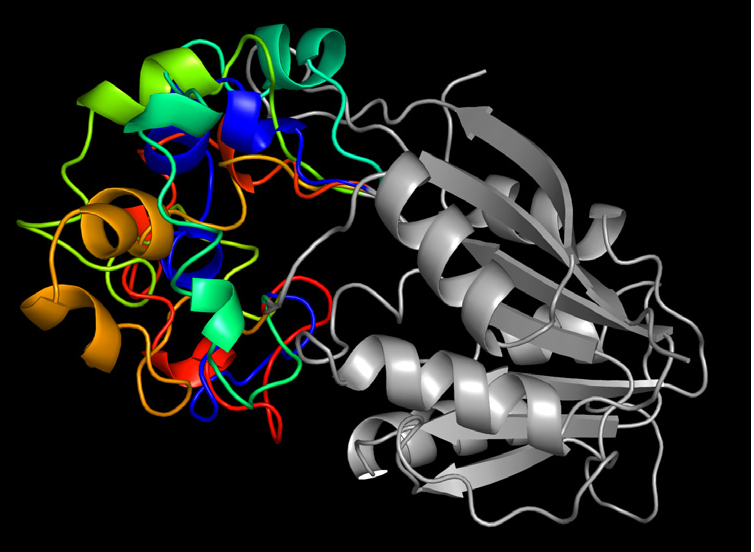
**Three-dimensional model of the HEN1 catalytic domain**. Superposition of five alternative models obtained by re-folding the insertion (aa 829–859) with ROSETTA. The homology-modeled core is in grey, five variants of the region modeled de novo are shown in different colors.

The refined alignment between HEN1 and the templates is shown in Figure 6; the corresponding model obtained the average VERIFY3D score of 0.352. Inspection of the quality of the local structures using the COLORADO3D server [39] revealed only one large region of poorly scoring residues: the insertion comprising aa 829–858 (data not shown). This region is known to be variable in RFM MTases (both on the sequence and the structure level) and in the structures of many small-molecules solved to date it was found to form a substrate-binding pocket. (J.M.B., L. Aravind, E.V. Koonin, unpublished data). Since template-based modeling of this insertion produced unsatisfactory models, we decided to model it *de novo*, using ROSETTA [40,41]. The well-scored parts of the model (aa 694–828 and 860–911) were kept unchanged, while the region 829–859 was allowed to re-fold, using the ROSETTA scoring function to identify physically sound, low-energy conformations (see Methods for details). Figure 7 shows the superposition of representatives of the five largest clusters (corresponding to the largest free-energy minima), obtained from the analysis of 8000 ROSETTA decoys. The coordinates in the PDB format, both for the initial model and for the alternative models generated with ROSETTA are available as supplementary data [see Additional files 1, 2, 3, 4, 5]. Although the conformations of the region modeled de novo shows substantial differences, in all models they assume a partially helical structure, which forms a wall of the potential substrate-binding site (see below). All analyzes of sequence-structure-function relationship described below, such as the mapping of residue conservation onto the structure were carried out for all five models. The results were qualitatively similar, hence for the sake of clarity we present here the analysis for only the model with the best Verify3D score 0.359.

### Model-based identification of amino acid residues important for substrate-binding and catalysis

**Figure 8:**
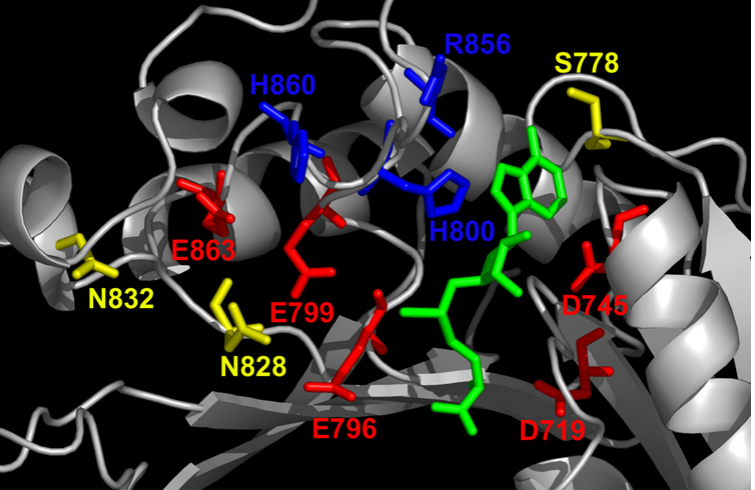
**Predicted functionally important residues mapped onto the model of HEN1 CTD (ROSETTA model 1)**. The AdoMet moiety is shown in the wireframe representation (green). Predicted AdoMet-binding residues are colored in yellow, catalytic residues are colored in red, RNA-binding residues are colored in blue.

Analysis of the sequence alignment of HEN1 homologs (Figure 2) in the light of the model(s) reveals the functional role of conserved residues and suggests the potential mechanism of ligand-binding and catalysis (Figure 8). Relatively easy to infer from the sequence alignment alone is the binding site for the methyl group donor S-adenosyl-L-methionine (AdoMet), which is strongly conserved in nearly all members of the RFM superfamily [42]. In HEN1 it comprises residues from motif I and the Gly-rich loop (peptide 721-GCGSG-725), which provides the structural framework for the binding pocket, motif II – D745 predicted to coordinate the 2' and 3'-OH groups of the ribose moiety, and motif III – S778 predicted to coordinate the N6 group of the adenine moiety. On the other hand, the active site of RFM enzymes is typically conserved within families, but not necessarily between families. Different families often have different active sites, adequately to the requirements of the reaction mechanism. For MTases acting on large molecules such as nucleic acids, the substrate-binding site is even more difficult to predict, as it can vary greatly even between members of the same family [42]. In the absence of obvious close homologs with similar function (as it is in the case of HEN1), the structural context greatly helps to infer the role of residues from motifs IV-VIII.

**Figure 9:**
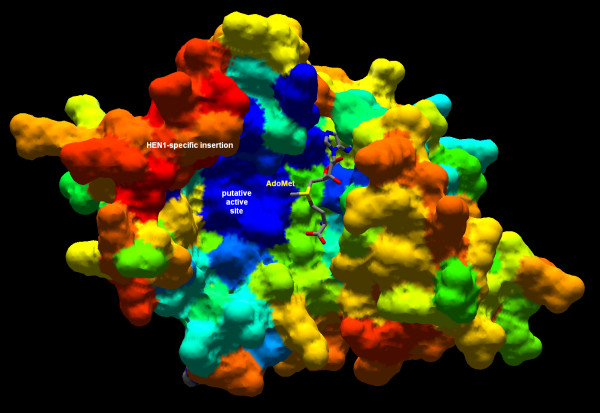
**Surface model of the HEN1 CTD (ROSETTA model 1) colored according to sequence conservation**. The spectrum of colors reflects the relative sequence conservation in the HEN1 family (blue – strongly conserved, through cyan – moderately conserved, to red – variable). The AdoMet moiety is shown in a wireframe representation.

**Figure 10:**
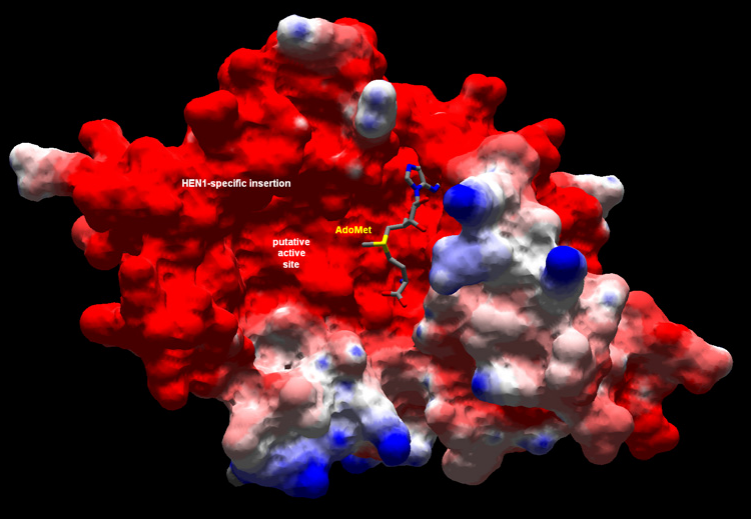
**Surface model of the HEN1 CTD (ROSETTA model 1) colored according to the distribution of the electrostatic potential**. The values of surface potentials are expressed as a spectrum ranging from -2 kT/e (deep red) to +2 kT/e (deep blue). The AdoMet moiety is shown in a wireframe representation.

The evolutionary information from the multiple sequence alignment of HEN1 homologs was mapped onto the surface of the modeled HEN1 structure using the CONSURF server [43]. Figure 9 reveals that conserved residues cluster together in the cofactor-binding pocket, as well as in the predicted substrate-binding site, formed by the common motifs IV, VI, VIII, and the additional motif specific to HEN1 (compare with Figure 2). Mapping of the electrostatic potential on the protein surface (Figure 10) reveals that the conserved cofactor and substrate-binding pockets are negatively-charged. On the other hand, there are adjacent positively charged patches on the HEN1 surface, which may be involved in the binding of the negatively charged RNA backbone, but they correspond to variable region around motifs III and X. If *A. thaliana *HEN1 binds its miRNA substrate using regions that are not conserved among its orthologs, then its substrate specificity may be different than that of other members of the HEN1 family. However, highly conserved residues E796, E799, H800 (motif IV), H828, H832 (motif VI), and R858, H860 (HEN1-specific insertion) around the putative active site suggest that at least the mechanism of methylation and probably also the details of interactions with the methylated part of the substrate (e.g. the ribose ring) may be very similar in all HEN1 homologs.

In agreement with the type of the template structures used, the spatial configuration of the C-terminal, surface-exposed part of motif IV of HEN1 at the bottom of the putative substrate-binding pocket is characteristic for small-molecule MTases. In particular, the peptide EhhEHh, (where h indicates a hydrophobic or aromatic residue) forms a small α-helix that is nearly perpendicular to all other secondary structure elements, which is commonly found in small molecule MTases, but thus far has not been identified in any nucleic acid MTase. Second, it conforms to the consensus sequence XhhEHh found in numerous small-molecule MTases, but is rather dissimilar to motif IV of typical MTases acting on nucleic acids (e.g. the (D/N/S)PP(Y/F/W/H) tetrapeptide of base MTases [44] or the DXXX motif of ribose MTases [31]). However, HEN1 contains an invariant Glu (E796) at the position commonly occupied by a carboxylate residue that participates in catalysis in nucleic acid MTases (e.g. by stabilization of the cofactor and the target in the preferred orientation and/or deprotonation of the substrate's attacking group), but is rarely present in small-molecule MTases.

The presumed catalytic pocket is also formed by conserved residues from motifs VI and X. Interestingly, the N-terminus of motif X in HEN1 reveals an invariant Arg residue (R701), which is located in a position similar to the invariant Lys residue in "orthodox" ribose MTases (e.g. K41 in VP39 or K38 in RrmJ). On the other hand, the surface-exposed C-terminal end of motif VI (located on the β-strand next to motif IV) is characterized by the pattern TPNXE(F/Y)N, which bears no similarity to its counterparts in other MTase families. In particular, it does not contain a Lys residue conserved and essential for catalysis in "orthodox" ribose MTases (e.g. K175 in VP39), which is proposed to position the hydroxyl oxygen toward the cofactor methyl group [45,46]. Thus, the K-D-K triad of residues from motifs X, IV, and VI found in "orthodox" ribose MTases [29,45] is definitely not conserved in the HEN1 family, although there is certain resemblance between the chemical character of invariant residues K175/D138 in VP39 and R701/E796 in HEN1.

Comparison of the putative active site of HEN1 with the ribose MTases from the SPOUT superfamily is even more difficult, as these proteins exhibit different folds and by definition do not share any homologous residues. The catalytic mechanism of SPOUT MTases is also much less understood than the mechanism of the RFM superfamily members, in part because of the lack of structural information on enzyme-substrate interactions. Nonetheless, several residues identified by the analysis of crystal structures and multiple sequence alignments have been found to be indispensable for the ribose MTase activity [47-49]. In particular, it has been proposed that the invariant Arg residue from one subunit in the SPOUT dimer (e.g. R145 in AviRb from *S. viridochromogenes*) is involved in steering the 2'-OH group of the target ribose towards the cofactor [48,49], in analogy to K175 in VP39 [46]. Here, we predict an analogous role also for R701 in HEN1. Important for the catalysis in SPOUT MTases are also two Asn residues (N139 and N262 in AviRb) that probably make contacts with the base of the methylated nucleoside. This role could be fulfilled by T826 and/or N828 from motif VI in HEN1.

Summarizing, we predict that the active site of HEN1 comprises R701 that orients the target hydroxyl group, E796 that stabilizes the cofactor and/or aids in deprotonation of the attacking oxygen atom. Other invariant or highly conserved residues of HEN1 such as T826 and/or N828 may be involved in binding of other regions of the substrate miRNA molecule (Figure 8). These predictions can be tested by site-directed mutagenesis of the respective residues.

## Conclusion

It is remarkable that the predicted catalytic pocket of HEN1 is different from the "K-D-K" active site triad of known ribose 2'-*O*-MTases from the RFM superfamily, e.g. VP39, RrmJ, or fibrillarin [29,31,45] even though these proteins share the three-dimensional fold with the HEN1 CTD. The active site of HEN1 (as well as of the RrmJ-related MTases) is of course also different from the active site of ribose 2'-*O*-MTases that belong to the unrelated the SPOUT superfamily, e.g. TrmH [49]. This suggests that ribose MTases evolved independently at least 3 times. Such independent origin of a particular type of MTase has been postulated also for enzymes that generate m^7^G in mRNA, rRNA, and tRNA [30,50,51], m^1^G in rRNA and different positions of tRNA [52-54], and m^2^G in different positions of tRNA [55,56]. Thus, convergent evolution of the reaction specificity appears to be very frequent among RNA MTases. Unfortunately, thus far crystal structures of enzyme-substrate complexes are not yet available for comparison of any of these apparent functional analogs among base MTases. Among ribose MTases, only a crystal structure of a VP39-RNA complex [57] is available, which has served as a template for functional analyses of other members of the RrmJ/fibrillarin variety [29,58], as well as unbound forms of structurally unrelated but functionally analogous enzymes from the SPOUT superfamily (e.g. [47,48]). Hence, until a high resolution structure of HEN1 or one of its homologs is obtained (preferably as a co-crystal with the RNA substrate), our model will serve as a convenient platform to study sequence-structure-function relationships in this enzyme and its relation to other MTases.

Our analyses reveal that HEN1 shares a number of structural features and most likely a closer phylogenetic origin with small-molecule MTases rather than with other known RNA MTases. The phylogeny of HEN1-CTD suggests that the ancestor of this protein family appeared already before the divergence of plants and animals/fungi, by duplication and subfunctionalization of a small-molecule MTase similar to UbiG. Perhaps the ancient HEN1-CTD has been transferred to Eukaryota by horizontal gene transfer from a bacterium.

It remains to be determined if the additional region present only in the plant members of the HEN1 family and composed of the DSRM domain, La-like domain, unknown central domain, and PPI-like domain, is essential for the MTase specificity for the miRNAs and what is the exact role of the individual domains. It is interesting to note that this extension is present only in HEN1 from *A. thaliana *and *O. sativa *and not in HEN1 orthologs from other organisms. It can be speculated that DSRM and La-like domains may be responsible for substrate binding by the orthodox HEN1 from plants. So far, no miRNAs have been identified in Bacteria. Besides, in miRNAs from *C. elegans *or *D. melanogaster *no 2' or 3'-methylation was found [11]. This suggests that the non-plant orthologs of HEN1 may be involved in methylation of some other substrates, which is particularly relevant given that HEN1 has apparently evolved from small-molecule MTases. Functional characterization of the short HEN1 orthologs, especially identification of their preferred substrates, and mutagenesis of the putative RNA-binding domains of plant HEN1 delineated in this work may shed the light on the evolution of specificity determinants in this interesting family of enzymes. It would be exciting to elucidate the evolutionary pathway leading from a small-molecule MTase to a nucleic acid MTase.

## Methods

### Sequence database searches

The BLAST family of algorithms [15,59] were used to search the non-redundant version of current sequence databases (nr), the publicly available complete and incomplete genome sequences, and the EST (expressed sequence tag) database at the NCBI [60]. Fragments of amino acid sequences (especially putative translations of the DNA sequences) were assembled into contiguous pieces using the sequence of *A. thaliana *HEN1 (GI 15638615) as a guide. The putative splicing sites were verified in reciprocal BLAST searches against the database comprising sequences of HEN1 homologs. All sequences were subsequently realigned using MUSCLE [24]. Manual adjustments were introduced into the multiple sequence alignment (MSA) based on the BLAST pairwise comparisons, secondary structure prediction, and results of the fold-recognition analyses (see below).

The final alignments were used to generate a set of query profile HMMs using HHmake from the HHsearch package [25]. The profile HHMs corresponding to all COG, KOG [61], PFAM [62], PDB70 [63], and CDD [17] entries were downloaded from the home site of HHsearch [64]. Comparison of the profile HMMs (sequence+structure) was carried out using HHsearch [25], with default parameters.

### Sequence clustering

To visualize pairwise similarities between and within protein families we used CLANS (CLuster ANalysis of Sequences), a Java utility that applies version of the Fruchterman-Reingold graph layout algorithm [27]. CLANS uses the P-values of high-scoring segment pairs (HSPs) obtained from an N × N BLAST search, to compute attractive and repulsive forces between each sequence pair in a user-defined dataset. Three dimensional representation is achieved by randomly seeding sequences in space. The sequences are then moved within this environment according to the force vectors resulting from all pairwise interactions and the process is repeated to convergence.

### Phylogenetic analyses

The refined multiple sequence alignment was used to calculate the phylogenetic tree of the HEN1 family using SplisTree [65], which employs the split decomposition model developed by Bandel and Dress [28]. The number of amino acid replacements per sequence position in the alignment was estimated using the JTT model [66]. Aiming at the determination of the sampling variance of the distance values, 1000 bootstrap resampling of the alignment columns was exerted.

### Protein structure prediction

Prediction of domains in the primary structure was carried out using the NCBI Conserved Domain Search utility [67]. Prediction of secondary structure, protein order/disoreder, solvent accessibility, and tertiary fold-recognition was carried out via the GeneSilico meta-server gateway (see [18] and [68] for details). Secondary structure prediction was predicted using PSIPRED [69], PROFsec [70], PROF [71], SABLE [72], JNET [73], JUFO [74], and SAM-T02 [75]. Protein disorder was predicted using PONDR [76] and DISOPRED [37]. Solvent accessibility for the individual residues was predicted with SABLE [72] and JPRED [77]. The fold-recognition analysis (attempt to match the query sequence to known protein structures) was carried out using FFAS03 [32], SAM-T02 [75], 3DPSSM [78], INBGU [79], FUGUE [80], mGENTHREADER [33], and SPARKS [34]. Fold-recognition alignments reported by these methods were compared, evaluated, and ranked by the Pcons server [35].

### Homology modeling

The alignments between the sequence of HEN1 and the structures of selected templates (members of the RFM fold identified by Pcons) were used as a starting point for modeling of the HEN1 CTD tertiary structure using the "FRankenstein's Monster" approach [36], comprising cycles of model building by MODELLER [81], evaluation by VERIFY3D [82] via the COLORADO3D server [39], realignment in poorly scored regions and merging of best scoring fragments. The positions of predicted catalytic residues and secondary structure elements were used as spatial restraints. This strategy has previously helped us to build accurate, experimentally validated models of other RNA MTases, including 16S tRNA:2'-OH MTase Trm7p [83], sno/snRNA:cap hypermethylase Tgs1 [84], tRNA:m^5^C MTase Trm4p [85], tRNA:m^1^A MTase TrmI [86], tRNA:m^2^G MTases from Archaea [56] and Eukaryota [87], and tRNA:m^7^G MTase TrmB [51]. Here, the refined comparative model comprised regions that could not be aligned to any of the templates and obtained unacceptably low scores in all models. Thus, they were re-modeled using a mixed "comparative/de novo" protocol, which has been successfully applied in the recent CASP6 competition to accurately model a variety of different proteins [88].

### De novo modeling

The insertion between motifs VI and VII (aa 829–858) was modeled de novo using ROSETTA [40] in the context of the rest of the HEN1 CTD modeled by homology (and kept invariant during modeling of the insertion). Briefly, fragment selection based on profile-profile and secondary structure comparison with the ROSETTA database was performed and 3 and 9 amino acids fragment lists were generated for the re-modeled regions. Fragment assembly was performed with default options and medium level of side chains rotamers optimization. The set of 8000 preliminary models (decoys) was clustered and representatives of 5 largest clusters were selected as the final structures.

## List of abbreviations

aa, amino acid(s); bp, base pair(s); CTD, C-terminal domain; DCL1, Dicer-like protein 1; DSRM, double-stranded RNA-binding motif; e, expectation; FKBP, FK506-binding protein; MTase, methyltransferase; miRNA, microRNA; ORF, product of an open reading frame; PPIase, peptidyl prolyl cis-trans isomerase; RFM, Rossmann-fold MTase.

## Authors' contributions

KLT carried out sequence database searches, phylogenetic analyses, and homology modeling, identified functionally important residues, prepared figures and participated in interpretation of the data and writing of the manuscript. AO carried out de novo modeling with ROSETTA and participated in preparation of the figures. JMB coordinated the whole study, participated in all analyses, interpretation of the data and drafted the manuscript. All authors have read and accepted the final version of the manuscript.

## Supplementary Material

Additional File 1 1^st ^model of the HEN1 CTD (aa 694–911) in the Protein Data Bank format. A representative of the largest cluster of decoys obtained after re-folding the insertion (aa 829–859) using ROSETTA. Click here for file

Additional File 2 2^nd ^model of the HEN1 CTD (aa 694–911) in the Protein Data Bank format. A representative of the 2^nd ^cluster of decoys obtained after re-folding the insertion (aa 829–859) using ROSETTA. Click here for file

Additional File 3 3^rd ^model of the HEN1 CTD (aa 694–911) in the Protein Data Bank format. A representative of the 3^rd ^cluster of decoys obtained after re-folding the insertion (aa 829–859) using ROSETTA. Click here for file

Additional File 4 4^th ^model of the HEN1 CTD (aa 694–911) in the Protein Data Bank format. A representative of the 4^th ^cluster of decoys obtained after re-folding the insertion (aa 829–859) using ROSETTA. Click here for file

Additional File 5 5^th ^model of the HEN1 CTD (aa 694–911) in the Protein Data Bank format. A representative of the 5^th ^cluster of decoys obtained after re-folding the insertion (aa 829–859) using ROSETTA. Click here for file
